# Structuring lightweight concrete with core–shell Fe_3_O_4_@DFNS nanoparticles: a path to smart and durable cementitious materials

**DOI:** 10.1039/d5ra04695e

**Published:** 2025-09-17

**Authors:** Seyed Ali Abutorabi, Amin Honarbakhsh, Rahele Zhiani, Seyed Mojtaba Movahedifar, Mehdi Nobahari

**Affiliations:** a Department of Civil Engineering, Ne.C., Islamic Azad University Neyshabur Iran amin_honarbakhsh@iau.ac.ir; b Department of Chemistry, Ne.C., Islamic Azad University Neyshabur Iran Zhiani@iau.ac.ir r_zhiani2006@yahoo.com

## Abstract

The integration of functional nanostructures into lightweight concrete (LC) offers a promising solution to address its inherent drawbacks, including high porosity, shrinkage, and limited durability under aggressive environmental conditions. This study evaluates the effect of Fe_3_O_4_@DFNS nanoparticles on the workability, ultrasonic pulse velocity (UPV), and durability of lightweight concrete. Experimental results show significant improvements in compressive strength, resistance to chloride ion penetration, and thermal conductivity, while maintaining the lightweight properties of the concrete. In this study, a novel core–shell nanocomposite comprising magnetic Fe_3_O_4_ nanoparticles encapsulated within dendritic fibrous nanosilica (Fe_3_O_4_@DFNS) was shown to enhance the performance of LC, particularly for marine applications. The Fe_3_O_4_@DFNS system was synthesized *via* a co-precipitation-sol–gel approach, combining magnetic dispersibility with high surface area and hierarchical porosity. Various dosages (0.05–0.35 wt%) were incorporated into LC mixes and evaluated through rheological, mechanical, thermal, and microstructural analyses. The optimal dosage (0.25 wt%) significantly improved workability and compressive strength by 30%, while reducing water absorption, porosity, and drying shrinkage. Durability assessments revealed a substantial enhancement in resistance to chloride ion ingress. Rapid chloride penetration test (RCPT), Fick's second law diffusion modeling, and chloride profiling confirmed over 45% reduction in penetration depth. Additional cyclic wetting–drying in artificial seawater showed 71% lower mass loss. Coupled FEM simulations validated improved stress distribution and delayed cracking, while X-ray CT revealed macro/micro void distribution and TEM confirmed the nanostructural features, together evidencing microstructural refinement. Furthermore, Fe_3_O_4_@DFNS reduced thermal conductivity by ∼20%, indicating potential for energy-efficient construction. The multifunctional behavior of this nanocomposite demonstrates its effectiveness as an intelligent additive for long-term durability and structural performance in coastal and marine-grade concrete.

## Abstract 2

The integration of functional nanostructures into lightweight concrete (LC) offers a promising solution to address its inherent drawbacks, including high porosity, shrinkage, and limited durability under aggressive environmental conditions.

## Graphical abstract

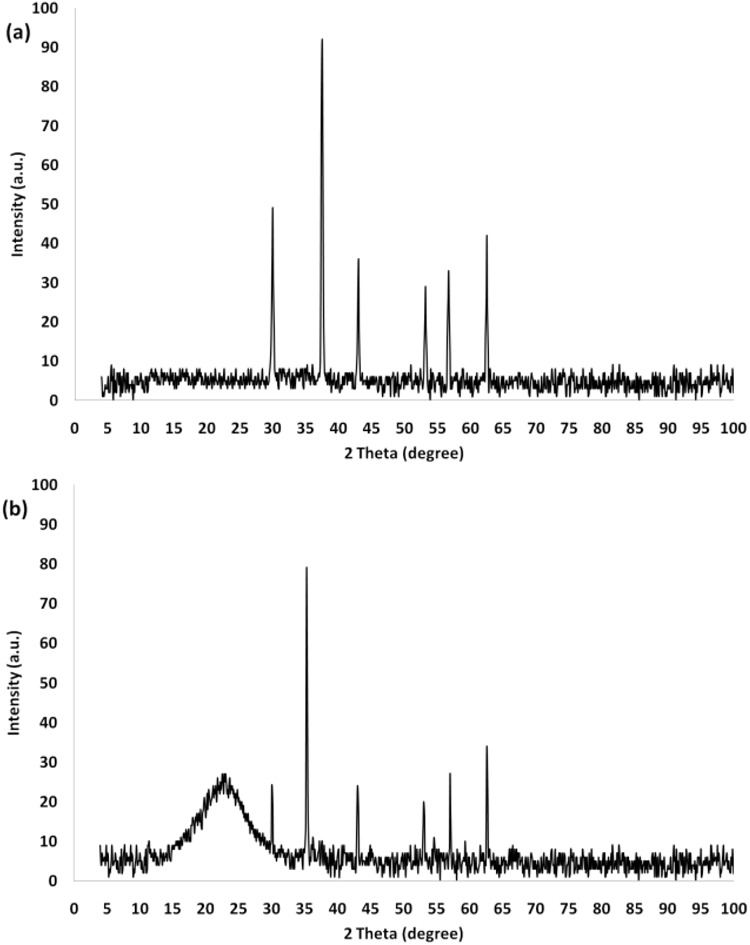

## Introduction

Lightweight concrete has become a vital material in the construction industry.^1^ Its use and range of applications have grown substantially over time. In many cases, lightweight concrete offers greater efficiency and cost-effectiveness compared to traditional building materials.^2^ Generally, lightweight concrete is characterized by its reduced weight and excellent flowability, making it adaptable for various applications.^3,4^

Despite its growing popularity, lightweight concrete has several drawbacks. These include limited water absorption capacity, higher drying shrinkage, reduced durability, and an increased susceptibility to cracking.^5^ These limitations arise from the material's porous internal structure especially its interconnected pore network which restricts its broader application in construction. One of the most severe durability concerns in lightweight concrete arises in marine and coastal environments, where aggressive chloride ions from seawater and airborne salt can penetrate the porous matrix, initiating corrosion of embedded steel reinforcements and deteriorating the overall structural integrity.^6^ In such environments, the demand for lightweight yet chloride-resistant concrete is growing, particularly for use in precast marine panels, offshore structures, and coastal infrastructure. Enhancing chloride resistance through nanostructured modifications has therefore become a critical direction in next-generation concrete development.^7^ Mohd Zamzani *et al.* showed that as the density of lightweight concrete decreases, the number of larger voids significantly increases. Furthermore, lightweight concrete exhibits lower ultrasonic pulse velocity and becomes more prone to cracking at reduced densities.^8^

Concrete materials are considered among the earliest applications where nanotechnology could drive significant future advancements.^9–11^ Research suggests that incorporating dense components can strengthen connections and reduce foam permeability. As a result, nanoscale particles such as silica, starch, and alumina have been employed to enhance the performance of preformed foams.^12–14^ Studies on large-scale particle-stabilized foams have shown no bubble growth for up to four days.^15^ Incorporating suitable nanoparticles can significantly improve the stability and overall performance of the cementitious matrix in lightweight concrete.^16–19^

To address these challenges, nanotechnology has emerged as a promising avenue in cement-based materials. Nanoparticles such as nanosilica and alumina have demonstrated the potential to refine pore structures, enhance hydration, and strengthen the cement matrix. Among these, dendritic fibrous nanosilica (DFNS) stands out due to its high surface area, radial fibrous morphology, and excellent dispersion in aqueous systems. Unlike conventional mesoporous silica, DFNS maintains structural integrity under modification and facilitates better chemical interaction with the cementitious phase. The use of arborescent filamentous nanomaterials enables the creation of nanostructured particles with a vast surface area. This extensive surface area is due to the presence of fibers rather than pores. The open and filamentous structure of DFNS allows for the integration of a significant volume of material without substantially reducing the external surface. Consequently, the increased accessibility of DFNS sites enhances surface interactions.^20–23^ This enhancement is linked to better dispersion enabled by the catalyst's fibrous core structure. The close interaction between molecular entities and quasi-particles adsorbed on DFNS fibers promotes effective engagement between induced charges and reactants.^24,25^

In this study, a novel nanostructure, Fe_3_O_4_@DFNS, is introduced, in which a magnetic Fe_3_O_4_ nanoparticle forms the core and is surrounded by a fibrous DFNS shell. This core–shell architecture offers dual functionality: the Fe_3_O_4_ core provides magnetic properties that improve dispersion during mixing and reduce particle agglomeration, while the DFNS shell offers high surface reactivity and compatibility with cement hydration products. Despite the proven advantages of both components individually, no prior research has investigated the synergistic effect of Fe_3_O_4_@DFNS in lightweight concrete, particularly under marine exposure conditions. This highlights a critical research gap that our study aims to address. This work aims to evaluate the influence of Fe_3_O_4_@DFNS on the microstructure, porosity, water absorption, ultrasonic wave transmission, and drying shrinkage of lightweight concrete. The ultimate goal is to enhance its durability and develop smarter, more resilient construction materials.

## Experimental section

### Substances and methods

In this study, ordinary Portland cement (PO 52.5) procured from Fars Cement Company (Iran) was used as the primary binder. The formulation of the lightweight foam concrete (FC) mixtures consisted of Portland cement (PC), standardized fine sand, a foaming protein-based agent, FFO-NP, and distilled water. The cement complied with BS12 specifications, featuring a specific gravity of 3.18 and a Blaine fineness of 3312 cm^2^ g^−1^. Comprehensive details regarding the chemical composition and physical properties of the cement are provided in Table 1, with additional parameters summarized in Table 2. To reinforce the matrix, magnetite nanoparticles with particle sizes ranging between 53 and 73 nm and a purity exceeding 98.5% were utilized (Table 3). A total of seven FC mixtures were prepared with a target unit weight of 1000 kg m^−3^, each incorporating Fe_3_O_4_@DFNS at different weight fractions: 0.00 wt%, 0.10 wt%, 0.15 wt%, 0.20 wt%, 0.25 wt%, 0.30 wt%, and 0.35 wt%. The mix design was based on a fixed water-to-cement ratio of 0.40 and a sand-to-water ratio of 1 : 1.4, as detailed in Table 4.

**Table 1:** Molecular composition of PC

Substance	Na_2_O	K_2_O	Fe_2_O_3_	SO_3_	Al_2_O_3_	MgO	SiO_2_	CaO	LOI
Percentage (%)	0.18	1.03	3.49	3.52	5.71	6.31	22.03	54.22	3.67

**Table 2:** Physical properties of Portland cement (PC)

Characteristics	Percentage (%)
Compressive strength (28-day) (N mm^−2^)	51.6
Particular gravity	3.31
Setting time (initial/final)	171/119
Specific surface area (cm^2^ g^−1^)	3298

**Table 3:** Key characteristics of the protein-derived foam stabilizer

Factors	Features
Appearance	Pale brown
pH	5.0 ± 0.7
Alkali content	<4.71%
Congestion (g cm^−3^)	1.07 ± 0.04 kg L^−1^
Molar mass	239 g mol^−1^
Chloride content	<0.14%
Specific gravity	1.00
Dilution ratio	1 : 28

**Table 4:** Mix design proportions of foam concrete (FC)

Sample	Weight fraction of Fe_3_O_4_@DFNS (wt%)
A	0.0%
B	0.10%
C	0.15%
D	0.20%
E	0.25%
F	0.30%
G	0.35%

### General procedure for the preparation of Fe_3_O_4_ MNPs

The synthesis of Fe_3_O_4_ nanoparticles was carried out through a co-precipitation method with the following steps. Initially, 0.01 mol of FeCl_2_·4H_2_O and 0.03 mol of FeCl_3_·6H_2_O were dissolved in 200 mL of distilled water. To this solution, 1.0 g of polyethylene glycol (PEG, MW = 6000) was added as a stabilizing agent. Subsequently, sodium hydroxide (NaOH) solution was gradually introduced to increase and maintain the pH at 12–13, providing the alkaline conditions necessary for the precipitation and formation of Fe_3_O_4_ nanoparticles. Hydrazine hydrate (N_2_H_4_·H_2_O, 80%) was then added in varying amounts to the alkaline suspension to initiate reduction and nanoparticle formation. The reaction mixture was stirred at room temperature for 24 hours, with continuous monitoring and adjustment of the pH to remain within the 12–13 range. The resulting black Fe_3_O_4_ nanoparticles were collected and thoroughly washed with deionized water to remove unreacted species and byproducts.

### General procedure for the preparation of Fe_3_O_4_@DFNS NPs

TEOS (1.9 g) was dissolved in a mixture of 1-pentanol (3.2 mL) and cyclohexane (46 mL). An aqueous solution containing urea (1.9 g) and cetylpyridinium bromide (3.2 g) was prepared which had been acidified with Fe_3_O_4_ (635 mg) in 13 mL of deionized water and gradually added to the blend while stirring. The mixture was stirred continuously for 32 minutes at room temperature before being transferred to a Teflon-lined hydrothermal reactor and heated to 135 °C for 8 hours. The resulting silica was collected *via* centrifugation, washed with deionized water and acetone, and then dried in an oven.

### Preparation of lightweight concrete (FC) samples

To prepare the lightweight concrete (FC) samples, ordinary Portland cement (PO 52.5), standard fine sand, and distilled water were used in accordance with BS EN 206. A protein-based foaming agent was incorporated to generate the porous structure, and Fe_3_O_4_@DFNS nanoparticles were dispersed within the mix at various weight fractions (0.00%, 0.10%, 0.15%, 0.20%, 0.25%, 0.30%, and 0.35%). The base mix design was targeted to achieve a dry density of 1000 ± 30 kg m^−3^. Prior to mixing, the Fe_3_O_4_@DFNS nanoparticles were ultrasonicated in water for 15 minutes to ensure uniform dispersion. The mixing process consisted of the following steps: (1) dry mixing of cement and sand for 2 minutes. (2) Addition of Fe_3_O_4_@DFNS suspension and mixing for 2 minutes. (3) Addition of foaming agent solution followed by gentle mixing for 3 minutes. (4) The fresh mixture was poured into prismatic (100 × 100 × 500 mm) and cylindrical (*Φ*35 × 40 mm) molds without compaction to preserve the pore structure. All samples were demolded after 24 hours and cured at 23 ± 2 °C in a humidity chamber (>95%) for up to 60 days.

### Ultrasonic pulse velocity (UPV) test

The ultrasonic pulse velocity of the fiber-reinforced lightweight concrete (FC) specimens was assessed using a pair of transducers positioned in a direct transmission arrangement one as a transmitter (T) and the other as a receiver (R) mounted on opposite ends of each specimen, as depicted in Fig. 4. Prism-shaped concrete samples with dimensions of 100 × 100 × 500 mm were prepared for the test, in accordance with BS EN 12504-4 standards. The test involved emitting ultrasonic pulses through the specimen and recording the travel time using a digital device capable of accurately measuring pulse propagation velocity. UPV readings were taken at three curing ages: 10, 20, and 30 days. For each age interval, three specimens were tested, and the mean value of the measured pulse velocities was reported as the representative result.

1 UPV = *L*/*T*

*L* = space between the convertors (mm), *T* = transfer time (s).

### Congestion test

The vacuum immersion technique was employed to determine the sponginess of the FC material. Three cylindrical specimens, each with a diameter of 35 millimeters and a height of 40 millimeters, were selected from each batch of the FC mixture for testing. These specimens were dried in an oven at 110 °C for 96 hours to remove any moisture. The exterior surfaces of the specimens were carefully smoothed using sandpaper. After drying, the weight of the specimens was measured, and they were subjected to a vacuum treatment ranging from 80–100 kPa for 3 days. Following the vacuum process, the specimens were immersed in boiling water until fully submerged. Following vacuum saturation, the FC specimens underwent immersion under reduced pressure conditions for approximately 30 hours to ensure complete elimination of entrapped air within both the concrete matrix and the surrounding water. Upon completion of this degassing phase, the saturated specimens were removed from the immersion chamber and immediately weighed to evaluate moisture uptake and saturation behavior.

2 Total porosity (%) = (*W*_sat_ − *W*_dry_)/(*ρv*)

*ρ* = density of water, *v* = mass of the polished FC case.

### Water absorption test

The water absorption experiment was conducted successfully following the procedures outlined in BS 1881-122. The FC specimens were weighed and wiped to determine the weight when the exterior surface was saturated but dry. Afterward, the specimens were oven-dried for 32 hours, and their weight was recorded again:

3 Water absorption (%) = ((*W*_sat_ − *W*_dry_)/*W*_dry_) × 100%

*W*_sat_ = saturated exterior zone dry weight (kg), *W*_dry_ = oven-dried weight (kg).

### Drying shrinkage test

To monitor dimensional changes due to drying, spherical gauge caps were affixed to both ends of the FC prismatic specimens to enable accurate length variation measurements. The specimens were subjected to drying in both radial and axial orientations. The initial length (*l*_1_) was recorded immediately after demolding, ensuring that any residual moisture on the gauge caps was carefully removed to eliminate potential measurement errors. A precision length comparator with a resolution of 0.003 mm was used to perform the readings over a total duration of 60 days. Shrinkage data were collected at regular intervals, starting from day 5 up to day 60.

### Compressive strength test

The compressive strength of the lightweight concrete (FC) samples containing Fe_3_O_4_@DFNS nanoparticles was evaluated to determine the influence of nanoparticle addition on mechanical performance. The test was performed in accordance with ASTM C109 using cube specimens measuring 50 mm × 50 mm × 50 mm. Three specimens were tested for each mix design and curing age (7, 28, and 56 days), and the average value was reported. The specimens were demolded after 24 hours and cured in a humidity chamber at 95% RH and 23 ± 2 °C. The compressive strength was measured using a universal testing machine with a loading rate of 0.5 MPa s^−1^.

### Thermal conductivity test

To assess the thermal insulation performance of lightweight concrete (FC) incorporating Fe_3_O_4_@DFNS nanoparticles, thermal conductivity was measured using the guarded hot plate method, conforming to ISO 8302. This method provides a reliable evaluation of steady-state thermal conductivity in porous construction materials. Disc-shaped FC specimens with a diameter of 100 mm and thickness of 20 mm were prepared from each mix design (Samples A–G) after 28 days of curing. All samples were oven-dried at 105 ± 2 °C for 48 hours to remove residual moisture and stabilize their internal microstructure prior to testing. The samples were placed between two stainless steel plates held at fixed temperatures of 45 °C (hot plate) and 15 °C (cold plate) to create a unidirectional thermal gradient. The heat flux through the sample was measured continuously until thermal equilibrium was achieved, and the thermal conductivity was calculated using Fourier's law of heat conduction:*k* = *qd*/Δ*T*where: *k*: thermal conductivity (W m^−1^ K^−1^). *q*: heat flux (W m^−2^). *d*: specimen thickness (m). Δ*T*: temperature difference across the specimen (K).

### Chloride ion penetration resistance (RCPT)

To evaluate the chloride resistance of lightweight concrete (FC) in marine-representative conditions, the Rapid Chloride Penetration Test (RCPT) was conducted in accordance with ASTM C1202, which is a standard method for assessing concrete's ability to resist chloride ion ingress. Cylindrical specimens of FC (100 mm diameter × 50 mm height) were prepared and cured for 28 days under standard conditions. To simulate marine-like exposure, the samples were vacuum-saturated with deionized water for 24 hours prior to testing, ensuring full pore saturation comparable to tidal or splash zone conditions. The specimens were then placed between two compartments: one containing 3% NaCl solution (chloride source), and the other containing 0.3 N NaOH solution. A 60 V DC voltage was applied across the sample for 6 hours. The total charge passed through the specimen (measured in Coulombs) was recorded automatically using a data acquisition system. Lower Coulomb values indicate better resistance to chloride ion penetration, which directly correlates with longer service life in coastal or marine environments. This test setup and interpretation approach aligns with durability evaluation protocols for marine concrete applications such as port decks, piers, seawalls, and offshore structures. This methodology provides a quantitative basis for comparing the ionic transport behavior of Fe_3_O_4_@DFNS-modified concretes under realistic marine exposure conditions.

### X-ray computed tomography (CT) imaging for internal pore structure analysis

To obtain a three-dimensional, nondestructive visualization of the internal pore structure and spatial distribution of Fe_3_O_4_@DFNS within lightweight concrete (FC), X-ray computed tomography (CT) imaging was performed. This technique provides a high-resolution representation of void morphology, connectivity, and nanoparticle influence on microstructural uniformity. For this purpose, cylindrical specimens of FC (diameter: 25 mm; height: 50 mm) were extracted from samples cured for 28 days. Each specimen was dried at 50 ± 2 °C for 48 hours to remove surface moisture and prevent imaging artifacts. X-ray CT scans were performed using a high-resolution micro-CT system (*e.g.*, SkyScan 1173) with the following parameters: voltage: 90 kV, current: 88 μA, pixel size (voxel resolution): 14 μm, rotation step: 0.4°, total scan angle: 360°. Reconstruction of raw data was performed using NRecon software, and 3D rendering and segmentation of pore spaces were conducted in CTAn and CTVol programs.

### Vibrating sample magnetometry (VSM) analysis

To confirm the magnetic properties of Fe_3_O_4_@DFNS nanoparticles and evaluate their suitability for enhanced dispersion and multifunctionality in lightweight concrete, vibrating sample magnetometry (VSM) analysis was conducted. The VSM technique provides a quantitative profile of the magnetic response, including saturation magnetization, remanence, and coercivity. Approximately 20 mg of dried Fe_3_O_4_@DFNS nanopowder was placed in a standard nonmagnetic sample holder and tested at room temperature (25 ± 1 °C) using a VSM system (*e.g.*, Lakeshore 7400). The magnetic field was applied in a sweeping range from −10 000 Oe to +10 000 Oe to obtain the full hysteresis loop.

## Results and discussion

**Scheme 1:**
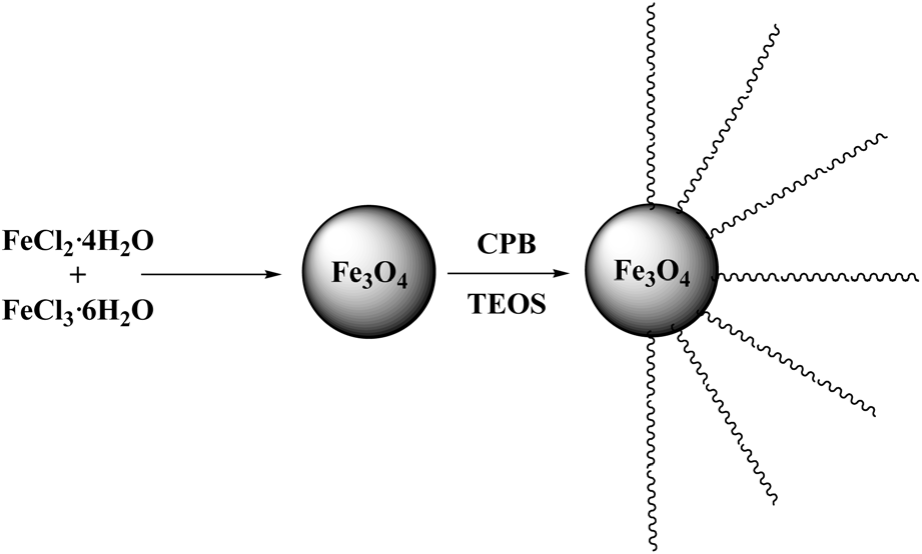
Schematic illustration of the merge for Fe_3_O_4_@DFNS.

The synthesization of the Fe_3_O_4_@DFNS nanocomposite is illustrated in Scheme 1. Initially, magnetite (Fe_3_O_4_) nanoparticles were synthesized *via* co-precipitation of ferrous and ferric salts in alkaline medium. These magnetic nanoparticles served as the core around which dendritic fibrous nanosilica (DFNS) was grown using a controlled sol–gel method. The TEOS precursor was hydrolyzed in the presence of surfactants and structure-directing agents, leading to the formation of radially oriented silica fibers around the Fe_3_O_4_ core. The abundant surface hydroxyl (Si–OH) groups on the DFNS shell facilitated stable encapsulation and structural compatibility within the cementitious matrix. This core–shell architecture provides both magnetic dispersibility and high surface area, making Fe_3_O_4_@DFNS an efficient multifunctional additive for concrete modification.

**Fig. 1:**
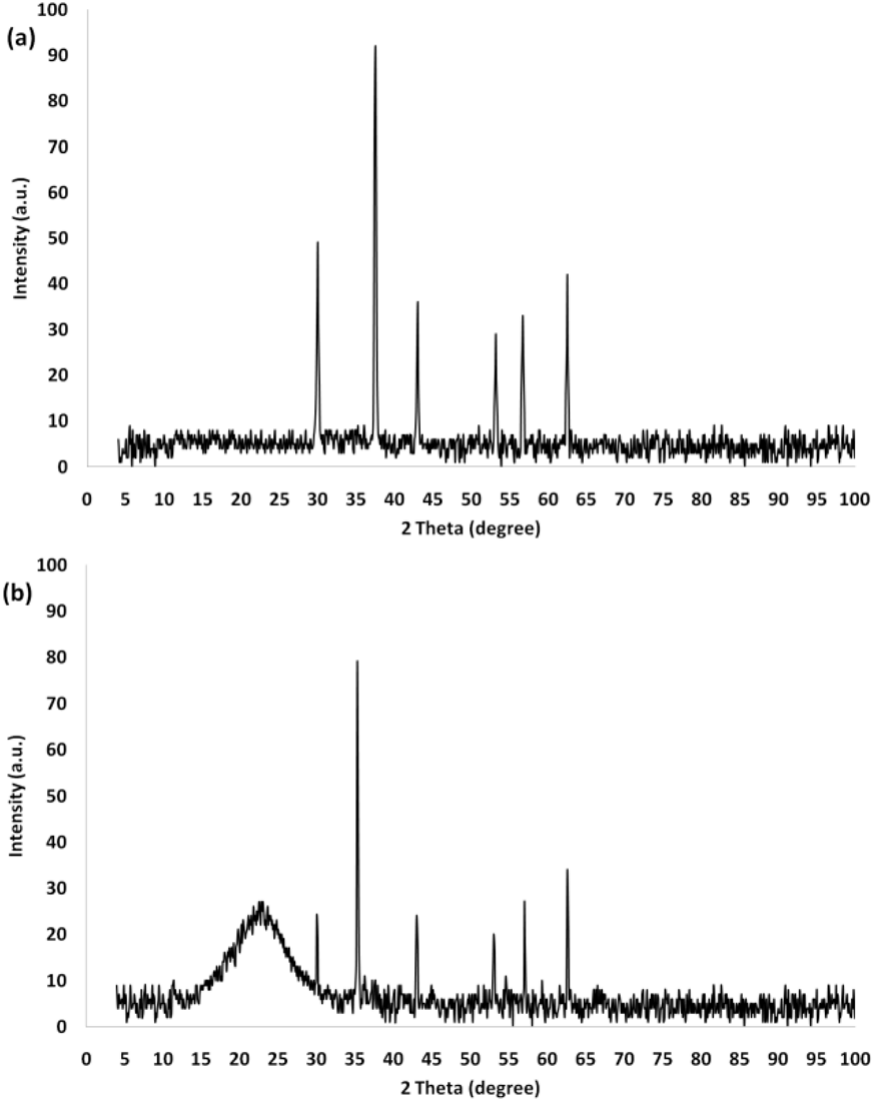
XRD patterns of Fe_3_O_4_ (a) and Fe_3_O_4_@DFNS (b).

The crystalline structures of Fe_3_O_4_ and Fe_3_O_4_@DFNS were characterized by X-ray diffraction (XRD), as shown in Fig. 1. The XRD pattern of pure Fe_3_O_4_ nanoparticles displayed distinct diffraction peaks at 2*θ* values of approximately 30.2°, 35.5°, 43.2°, 53.5°, 57.1°, and 62.7°, which correspond well to the (220), (311), (400), (422), (511), and (440) planes of the inverse spinel structure of magnetite (JCPDS card no. 19-0629). These reflections confirm the formation of well-crystallized Fe_3_O_4_ nanoparticles (Fig. 1a). In the Fe_3_O_4_@DFNS sample, similar peaks associated with the magnetite phase were observed, albeit with slightly reduced intensity due to the amorphous nature of the silica shell. A broad hump in the range of 20°–30° was also present, indicating the existence of non-crystalline silica from the DFNS coating. The preservation of Fe_3_O_4_ crystalline features alongside the DFNS background pattern confirms the successful formation of the core–shell structure without compromising the magnetic integrity of Fe_3_O_4_. These results affirm that the encapsulation of Fe_3_O_4_ within the DFNS matrix did not alter its crystallinity, while the DFNS layer contributed to structural stability and dispersion capability, which are crucial for its performance in cementitious applications (Fig. 1b).

**Fig. 2:**
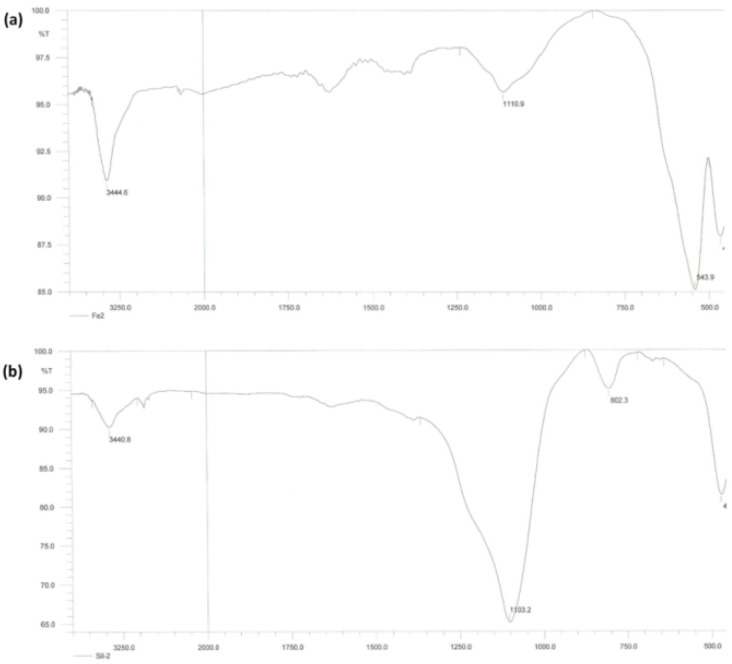
FT-IR spectra of (a) Fe_3_O_4_, and (b) Fe_3_O_4_@DFNS.

The successful synthesis of Fe_3_O_4_ and Fe_3_O_4_@DFNS nanoparticles was verified by FTIR spectroscopy, as presented in Fig. 2. In the spectrum of bare Fe_3_O_4_ (Fig. 2a), a broad absorption band around 3444 cm^−1^ corresponds to the O–H stretching vibrations from surface hydroxyl groups, while a distinct peak at 543 cm^−1^ is attributed to the Fe–O stretching mode of magnetite. After coating with DFNS, the FTIR spectrum of Fe_3_O_4_@DFNS (Fig. 2b) displayed a noticeable decrease in the Fe–O band intensity due to the silica shell coverage. Simultaneously, strong absorption bands appeared at 1103 cm^−1^ and 802 cm^−1^, corresponding to the asymmetric and symmetric stretching vibrations of Si–O–Si, respectively. These findings confirm the successful formation of the core–shell architecture, with Fe_3_O_4_ as the magnetic core and DFNS as the fibrous silica shell. The presence of Si–OH and Si–O–Si groups also supports the potential chemical compatibility and reactivity of the nanocomposite within cementitious matrices.

**Fig. 3:**
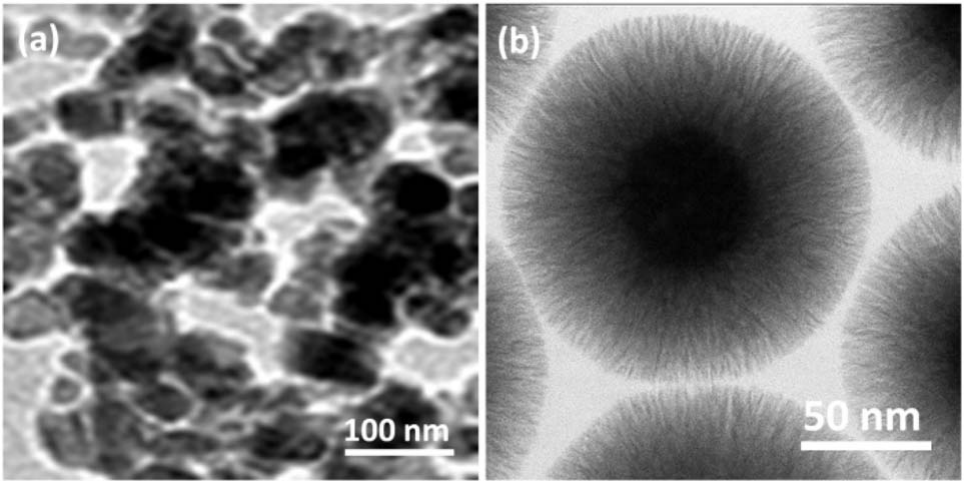
TEM images of Fe_3_O_4_ (a); and Fe_3_O_4_@DFNS (b).

The morphological features of Fe_3_O_4_ and Fe_3_O_4_@DFNS nanoparticles were analyzed using transmission electron microscopy (TEM), as shown in Fig. 3. The TEM image of Fe_3_O_4_ nanoparticles (Fig. 3a) reveals uniformly distributed spherical particles with smooth surfaces, indicative of well-crystallized magnetite. These particles exhibit moderate agglomeration and an average diameter in the range of 20–30 nm, consistent with previous reports on magnetite nanoparticles. In contrast, the Fe_3_O_4_@DFNS nanostructures (Fig. 3b) display a distinct core–shell architecture, where the dense Fe_3_O_4_ cores are encapsulated within a fibrous silica shell. The DFNS layer consists of radially oriented silica nanofibers, forming a dendritic morphology that extends uniformly outward from the magnetic core. The average diameter of the composite spheres is approximately 250–300 nm, with individual fiber thicknesses estimated at ∼8–10 nm. The high degree of monodispersity and the open wrinkled structure of the DFNS shell suggests the formation of accessible mesoporous channels. This hierarchical and porous configuration is advantageous for mass transport, nanoparticle dispersion, and chemical compatibility in cementitious matrices. Notably, the encapsulation of Fe_3_O_4_ within DFNS does not disrupt the spherical morphology, indicating that the synthesis process effectively preserves structural integrity during shell formation. Such architecture enhances both the dispersibility and functional performance of the nanomaterials when used in concrete applications, especially under aggressive environmental conditions such as chloride exposure.

**Fig. 4:**
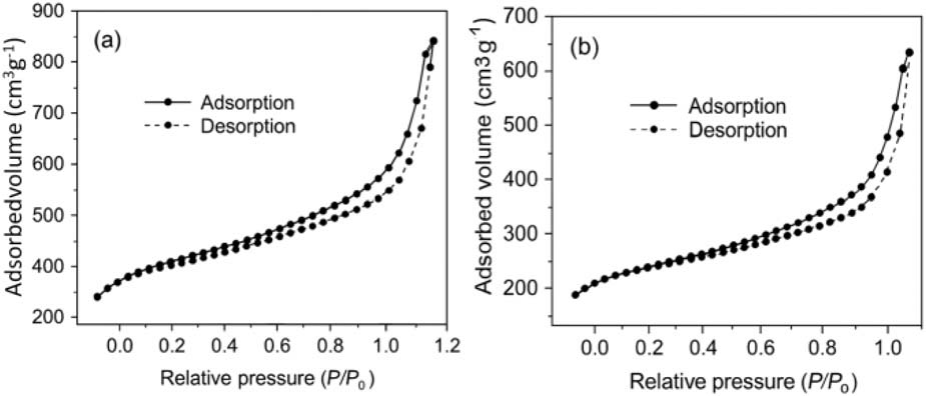
Adsorption–desorption isotherms of (a) DFNS, and (b) Fe_3_O_4_@DFNS.

Mesoporous-based materials frequently suffer from reduced surface accessibility, pore volume, and structural stability upon functionalization, as the incorporation of additional phases or molecules often blocks internal channels. This limitation can significantly hinder mass transport and decrease the overall efficiency of the modified material. To overcome this issue, DFNS was selected as a support due to its inherently open fibrous morphology and high surface area. When Fe_3_O_4_ nanoparticles were integrated into the DFNS matrix to form Fe_3_O_4_@DFNS, the resulting material retained the essential porous features of the silica shell. The nitrogen adsorption–desorption isotherms of Fe_3_O_4_@DFNS exhibited a characteristic type IV profile (Fig. 4), indicative of mesoporous behavior, which is consistent with previous reports on fibrous silica-based nanostructures. As summarized in Table 5, the incorporation of Fe_3_O_4_ nanoparticles into the core did not compromise the overall fibrous architecture or void volume of the DFNS shell. The preservation of this architecture enables efficient diffusion pathways and ensures the compatibility of Fe_3_O_4_@DFNS with aqueous and cementitious systems, making it a promising multifunctional additive for durability enhancement in concrete.

**Table 5:** Architectural metrics of DFNS, and Fe_3_O_4_@DFNS materials determined from nitrogen absorption experiments

Sample	Surface area (*S*_BET_, m^2^ g^−1^)	Pore volume (cm^3^ g^−1^)	Average pore diameter (nm)
DFNS	568	1.86	19.34
Fe_3_O_4_@DFNS	359	0.89	10.34

**Fig. 5:**
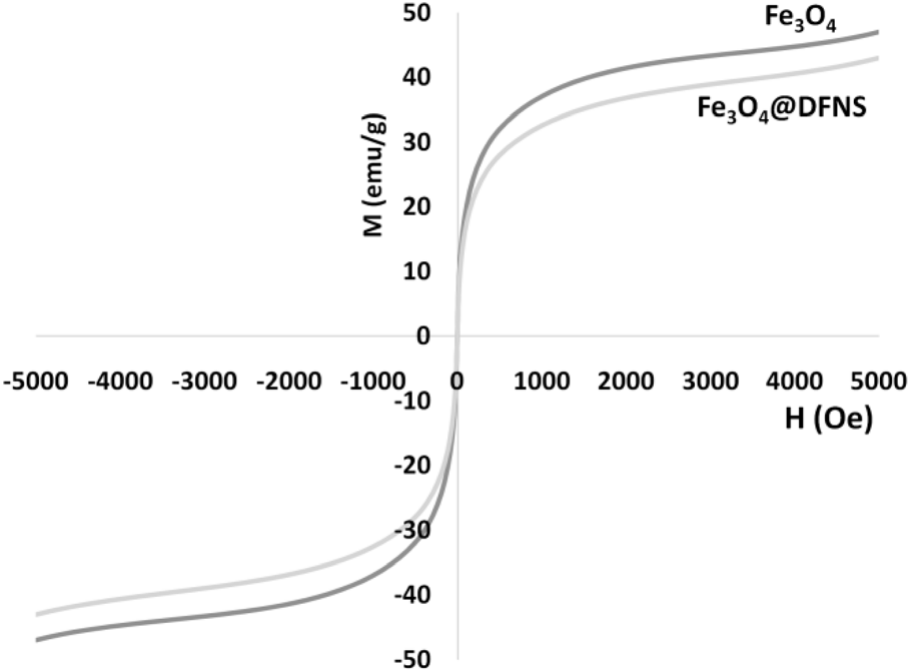
Room-temperature magnetization curves of the nanoparticles.

The magnetic hysteresis curve is shown in Fig. 5. The Fe_3_O_4_@DFNS sample exhibited a typical soft magnetic behavior with no remanent magnetization or coercivity, confirming superparamagnetic characteristics. Compared to pure Fe_3_O_4_ nanoparticles, the reduced saturation magnetization in Fe_3_O_4_@DFNS is due to the silica shell, which is nonmagnetic and dilutes the overall magnetic response. Despite this reduction, the value of 28.4 emu g^−1^ remains sufficient to enable: magnetic response during concrete mixing, enhancing dispersion and homogeneity, and potential field-triggered functionality in smart cementitious systems. The soft magnetic nature ensures that the particles do not aggregate post-mixing, supporting uniform distribution throughout the matrix. The VSM analysis confirms that Fe_3_O_4_@DFNS nanoparticles retain strong magnetic responsiveness while exhibiting superparamagnetic behavior due to their nanoscale core and silica encapsulation. This feature supports their dual role as a structural modifier and active functional additive in smart and durable lightweight concrete formulations.

Table 6 presents the volumetric content of Fe_3_O_4_@DFNS across different FC mixtures alongside their corresponding bulk dry densities. The target dry density for all mixes was set at 1000 kg m^−3^. It was critical to maintain a density differential of approximately 110–130 kg m^−3^ between the fresh (plastic) and dried states. According to standard guidelines, the allowable deviation from the target dry density ranged from ±30 kg m^−3^ for low-density fibre-cement systems (500–1400 kg m^−3^) to ±40 kg m^−3^ for higher-density formulations (1400–2100 kg m^−3^). Dry density measurements were conducted at curing ages of 7, 28, and 56 days. For all FC mixes, the observed values of dry compaction fell within the acceptable tolerance of ±40 kg m^−3^. For instance, the differences between the measured and target dry densities were recorded as ±12, ±15, ±21, ±22, ±24, ±26, and ±27 kg m^−3^ for samples A through G, respectively. These minor deviations confirm the consistency of the mix design and processing conditions. Notably, the final dry density played a significant role in influencing the mechanical strength and long-term durability of the FC, underlining its importance as a key performance parameter.

**Table 6:** Bulk dry density of FC mixtures

Specimen number	Dry density (kg m^−3^)/curing age (days)		
	7	28	56
Sample A	1013	1004	1002
Sample B	1016	1008	1003
Sample C	1020	1009	1005
Sample D	1022	1011	1007
Sample E	1025	1013	1009
Sample F	1026	1014	1010
Sample G	1028	1015	1012

**Fig. 6:**
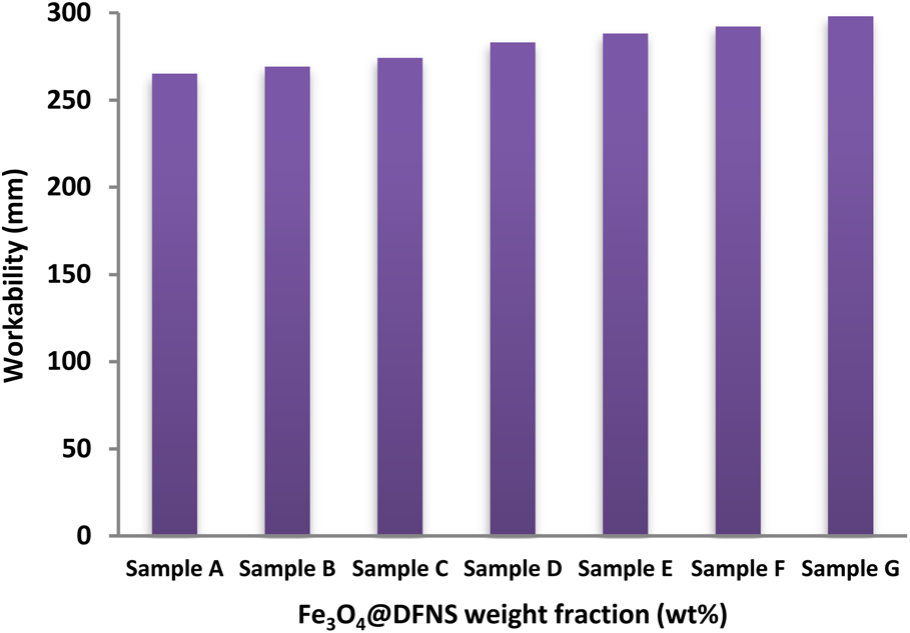
Effect of Fe_3_O_4_@DFNS content on the workability of foam concrete (FC).

In this study, the workability of fresh foam concrete (FC) mixtures was evaluated using the flow table spread test, following standard procedures to assess uniformity and flow behavior. The test aimed to determine the dispersion characteristics of the mixtures and detect any tendency toward segregation. The incorporation of Fe_3_O_4_@DFNS nanoparticles led to an increase in the viscosity of the liquid phase, which in turn delayed the mobility of cement fillers and improved overall mix stability. The results of the flow table test for all FC formulations are illustrated in Fig. 6. The control sample (Sample A) exhibited the lowest spread diameter at 264 mm, while the inclusion of Fe_3_O_4_@DFNS in Samples B through G progressively improved flowability, reaching a maximum of 298 mm in Sample G. This enhancement is primarily attributed to the hydrophobic nature of Fe_3_O_4_@DFNS, which increases the amount of free water in the system and promotes better cohesion among the mix components. Moreover, the low surface porosity of Fe_3_O_4_@DFNS reduces its interaction with the surrounding cementitious matrix, leading to decreased internal friction and increased slump flow. The nanoparticles also contribute to uniform air bubble formation, which enhances the structural stiffness of the fresh mixture without engaging in chemical interactions with the protein-based foaming agent. The limited surface activity of Fe_3_O_4_@DFNS allows for better dispersion and matrix flow, ultimately resulting in improved workability and homogeneity of the FC blends.

**Fig. 7:**
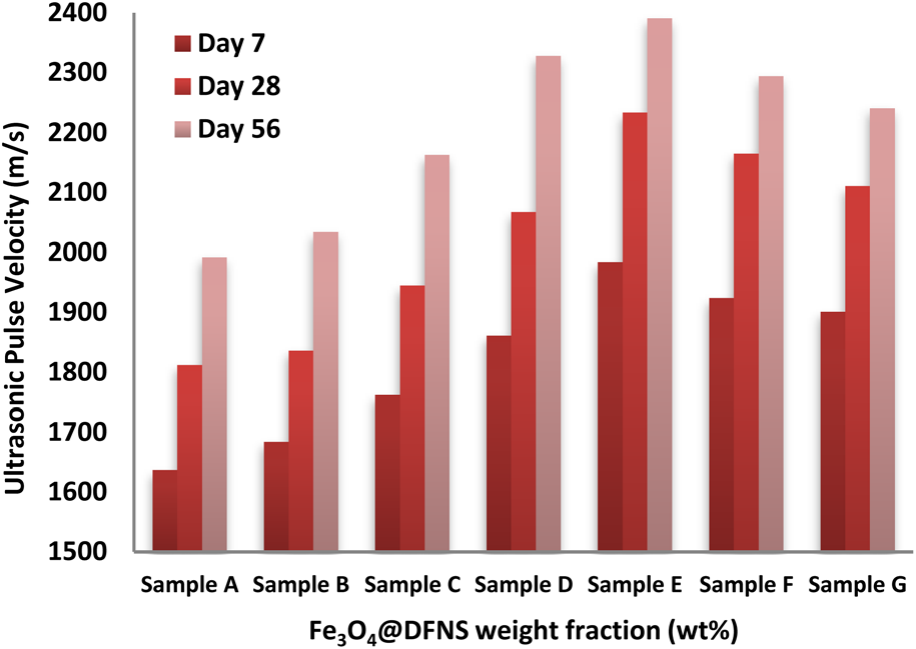
Ultrasonic pulse velocity of FC specimens containing different Fe_3_O_4_@DFNS dosages.

The ultrasonic pulse velocity (UPV) test was employed to evaluate the structural integrity and quality of the foam concrete (FC) mixtures by measuring the velocity of acoustic waves transmitted through the material. Higher UPV values are generally indicative of denser, more cohesive internal structures, while lower velocities typically suggest the presence of internal voids, microcracks, or poor compaction. The UPV results corresponding to various weight fractions of Fe_3_O_4_@DFNS are illustrated in Fig. 7. The recorded UPV values among the samples were relatively close, suggesting that large-scale voids were effectively minimized during specimen preparation. Notably, Sample E (containing 0.25 wt% Fe_3_O_4_@DFNS) demonstrated a substantial improvement in acoustic velocity, reaching 2234 m s^−1^ at 28 days, compared to 1812 m s^−1^ for the control specimen (Sample A)—an enhancement of approximately 23%. This improvement can be attributed to the nanoparticle-induced densification of the matrix, which reduced internal porosity and hindered the propagation of microcracks. The presence of Fe_3_O_4_@DFNS enhanced early-age hydration, leading to a more refined microstructure and improved acoustic continuity. However, further increases in nanoparticle content beyond the optimal dosage (Sample E) led to a decline in UPV values. In Sample F, the excessive inclusion of Fe_3_O_4_@DFNS resulted in particle agglomeration and uneven dispersion within the cement matrix, which interfered with hydration and reduced the speed of wave propagation. Despite this decline, Sample G still exhibited a higher UPV than the control, indicating that Fe_3_O_4_@DFNS, when well-dispersed, consistently contributes to structural improvement within the FC matrix.

**Fig. 8:**
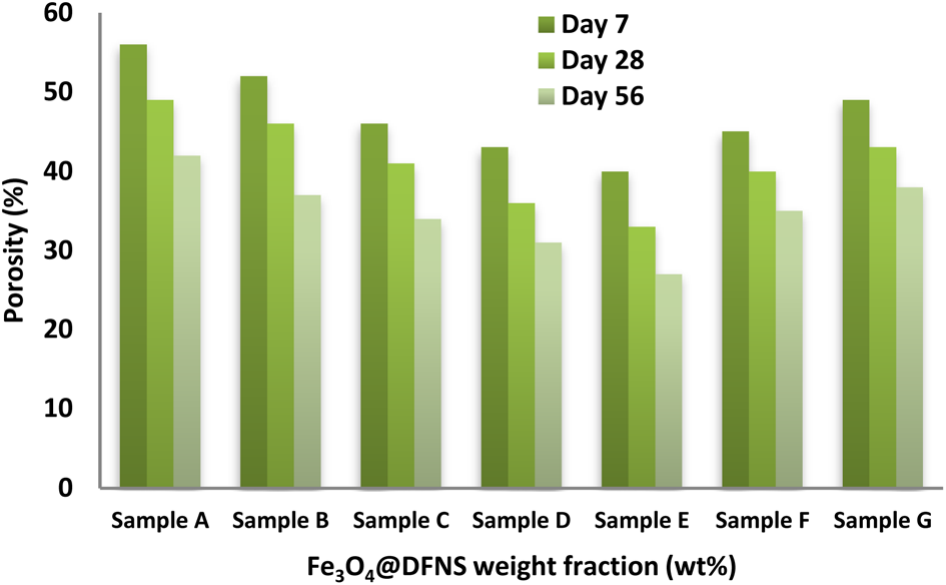
Effect of Fe_3_O_4_@DFNS content on the porosity of foam concrete (FC).

Fig. 8 presents the effect of varying Fe_3_O_4_@DFNS mass fractions on the porosity of foam concrete (FC). Among the tested samples, Sample E (0.25 wt%) exhibited the lowest porosity, showing a significant reduction of approximately 23% compared to the control (Sample A). After 28 days, the porosity of Sample E was recorded at 33%, in contrast to 49% for Sample A. This notable improvement is attributed to the enhanced hydration and microstructural densification facilitated by the uniformly dispersed Fe_3_O_4_@DFNS particles, which effectively filled internal voids. However, porosity began to increase in samples with Fe_3_O_4_@DFNS contents above the optimal threshold. This adverse trend is primarily linked to nanoparticle agglomeration, which reduces dispersion efficiency and limits void-filling potential. Additionally, the presence of Fe_3_O_4_@DFNS at higher dosages led to the formation of microcracks along the matrix boundaries, promoting localized fluid movement and increasing water diffusivity due to capillary dynamics. The variation in porosity and water transmission among samples with identical Fe_3_O_4_@DFNS loadings can be explained by heterogeneities in particle distribution and cement paste interaction. In contrast, the control mix (Sample A) exhibited a more porous and interconnected void structure, which compromised its durability and mechanical integrity. Overall, the inclusion of Fe_3_O_4_@DFNS contributed to the refinement of pore structure by reducing both the number and size of voids, improving particle packing, and strengthening the bond between sand and the cementitious matrix. Furthermore, the formation of blocked and narrower capillary pores reduced the overall permeability and absorption coefficient of the FC composite, indicating improved durability and structural efficiency.

**Fig. 9:**
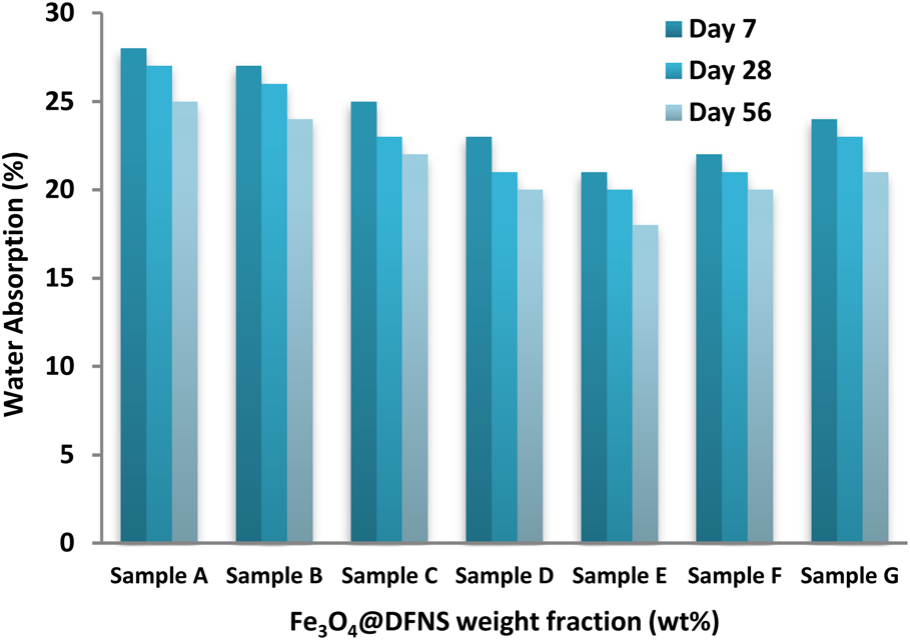
Moisture absorption of FC specimens with varying Fe_3_O_4_@DFNS dosages over time.

The moisture absorption behavior of the foam concrete (FC) specimens was evaluated at selected curing ages of 7, 28, and 56 days, with the results illustrated in Fig. 9. Among all mixtures, Sample A (control) exhibited the highest water uptake, recording absorption values of 28%, 27%, and 25% at the respective time intervals. The incorporation of Fe_3_O_4_@DFNS notably reduced moisture ingress in the FC matrix. In early curing stages, water uptake was predominantly influenced by larger capillary pores, whereas in later stages, finer pores governed absorption behavior. Sample E (containing 0.25 wt% Fe_3_O_4_@DFNS) showed the most significant improvement, reducing water absorption by approximately 18%, 20%, and 21% at 7, 28, and 56 days, respectively, compared to the control. This enhancement is attributed to the dual role of Fe_3_O_4_@DFNS as both a physical filler and a hydration promoter leading to reduced porosity and improved matrix continuity. The nanoparticles acted as micro-fillers, bridging voids and reinforcing the interfacial transition zone, thus impeding moisture ingress.

**Fig. 10:**
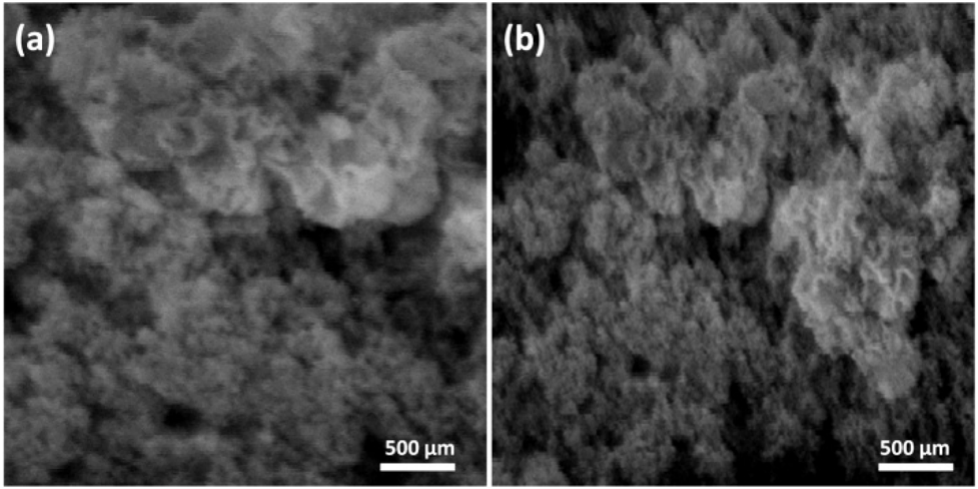
SEM micrograph control FC specimens (a); FC with 0.25% Fe_3_O_4_@DFNS (b).

Fig. 10a presents a Scanning Electron Microscopy (SEM) image of the control sample, revealing large interconnected voids throughout the matrix. Conversely, Fig. 10b depicts the microstructure of FC modified with Fe_3_O_4_@DFNS, where improved dispersion and agglomeration of the nanocomposite contributed to a denser and more homogeneous internal structure. The presence of Fe_3_O_4_@DFNS not only improved packing efficiency but also stimulated additional hydration reactions, further densifying the matrix. However, increasing the Fe_3_O_4_@DFNS content beyond 0.25 wt% led to diminishing returns. At higher dosages, nanoparticle agglomeration became more prominent, reducing their ability to penetrate and fill microvoids. This aggregation effect slightly increased water permeability due to incomplete pore obstruction, highlighting the importance of dosage optimization. In addition to its physical filler effect, DFNS contributes significantly through chemical and physicochemical interactions with cement hydration products. The high surface area and fibrous morphology of DFNS provide abundant nucleation sites for calcium silicate hydrate (C–S–H) gel, thereby accelerating hydration and promoting a denser microstructure. The surface silanol (Si–OH) groups of DFNS can bind Ca^2+^ ions released during hydration, facilitating stronger interfacial bonding between nanoparticles and the cementitious matrix. Moreover, the amorphous silica phase of DFNS exhibits pozzolanic activity by reacting with portlandite (Ca(OH)_2_), forming additional secondary C–S–H that further refines the pore structure and reduces permeability. These combined effects—nucleation of hydration products, ion exchange, pozzolanic reactivity, and microstructural densification—explain the observed improvements in mechanical strength, shrinkage resistance, and chloride durability of the Fe_3_O_4_@DFNS-modified lightweight concrete.^26,27^

**Fig. 11:**
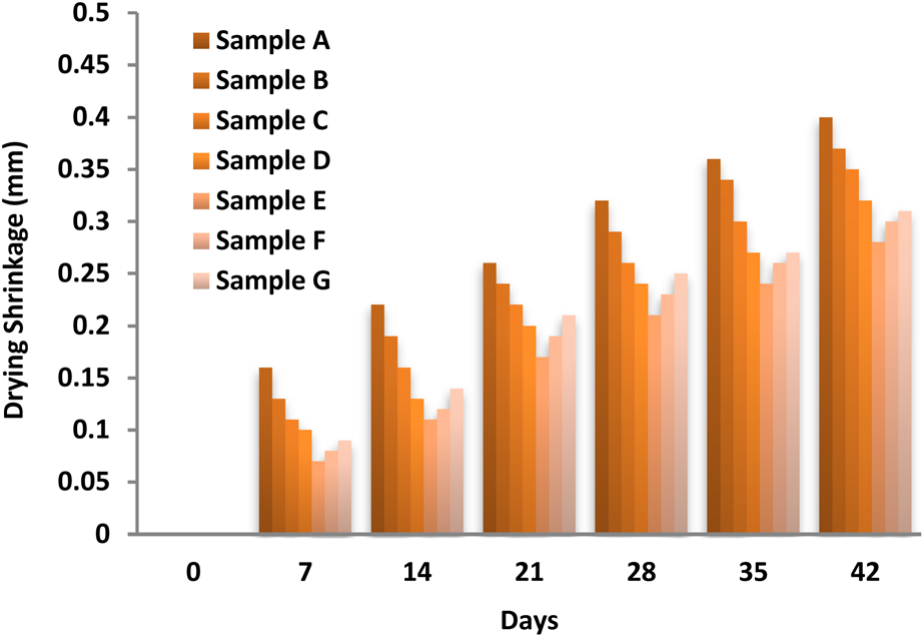
Drying shrinkage of FC specimens with varying Fe_3_O_4_@DFNS content.

Drying shrinkage in concrete refers to the volumetric reduction that occurs primarily due to moisture loss under low ambient humidity conditions. This phenomenon is one of the major contributors to crack formation in hardened concrete. As a rheological material, the FC binder exhibits both elastic and viscous deformation behavior, meaning that its shrinkage response involves time-dependent volumetric changes. Fig. 11 illustrates the evolution of drying shrinkage in FC mixtures containing different mass fractions of Fe_3_O_4_@DFNS. All samples showed progressive shrinkage over time; however, the control specimen (Sample A) experienced the highest degree of contraction. The incorporation of Fe_3_O_4_@DFNS significantly reduced drying shrinkage, with Sample E (0.25 wt%) demonstrating the most effective suppression of volumetric contraction. The improved performance is attributed to the ability of Fe_3_O_4_@DFNS to absorb and dissipate tensile energy generated during shrinkage, particularly at the interfacial transition zone between the nanoparticle surface and the surrounding matrix. This energy redistribution reduces localized tensile stress concentrations and delays the onset of microcracking. Additionally, the fibrous silica shell of DFNS promotes mechanical interlocking and nucleation of hydration products, further contributing to shrinkage resistance. However, when the Fe_3_O_4_@DFNS content exceeded the optimal dosage, shrinkage mitigation diminished. This was due to particle agglomeration and poor dispersion within the matrix, which led to a balling effect that compromised stress transfer and reduced the effectiveness of internal reinforcement. Therefore, a well-dispersed 0.25 wt% loading of Fe_3_O_4_@DFNS is identified as the optimal concentration for enhancing shrinkage resistance and improving dimensional stability in FC composites.

Table 7 shows the compressive strength results for FC samples containing 0.00% to 0.35% Fe_3_O_4_@DFNS. The control specimen (Sample A, 0.00%) exhibited compressive strengths of 8.2 MPa, 9.5 MPa, and 10.1 MPa at 7, 28, and 56 days, respectively. The inclusion of Fe_3_O_4_@DFNS significantly enhanced compressive strength across all curing ages. The optimum improvement was observed in Sample E (0.25%), which recorded strengths of 10.3 MPa (7 days), 12.6 MPa (28 days), and 13.2 MPa (56 days). This improvement is attributed to the nano-filler effect of Fe_3_O_4_@DFNS, which densifies the matrix by occupying microvoids and enhancing cement hydration. However, at higher concentrations (Samples F and G), a slight decline in strength was noted. This is likely due to nanoparticle agglomeration, which disrupted matrix homogeneity and created localized weaknesses. The Fe_3_O_4_ core contributes to uniform dispersion due to its magnetic response during mixing, preventing aggregation and facilitating better bonding with hydration products. The DFNS shell, with its high surface area, acts as a scaffold for calcium–silicate–hydrate (C–S–H) formation. Together, they promote a refined microstructure, reduce pore connectivity, and improve load transfer capability under compressive loading.

**Table 7:** Compressive strength of lightweight concrete samples containing varying weight fractions of Fe_3_O_4_@DFNS nanoparticles at 7, 28, and 56 days

Sample	7-Day strength (MPa)	28-Day strength (MPa)	56-Day strength (MPa)
A	8.2	9.5	10.1
B	8.9	10.7	11.3
C	9.4	11.5	12.1
D	9.9	12.1	12.7
E	10.3	12.6	13.2
F	9.8	11.9	12.3
G	9.5	11.4	11.8

**Fig. 12:**
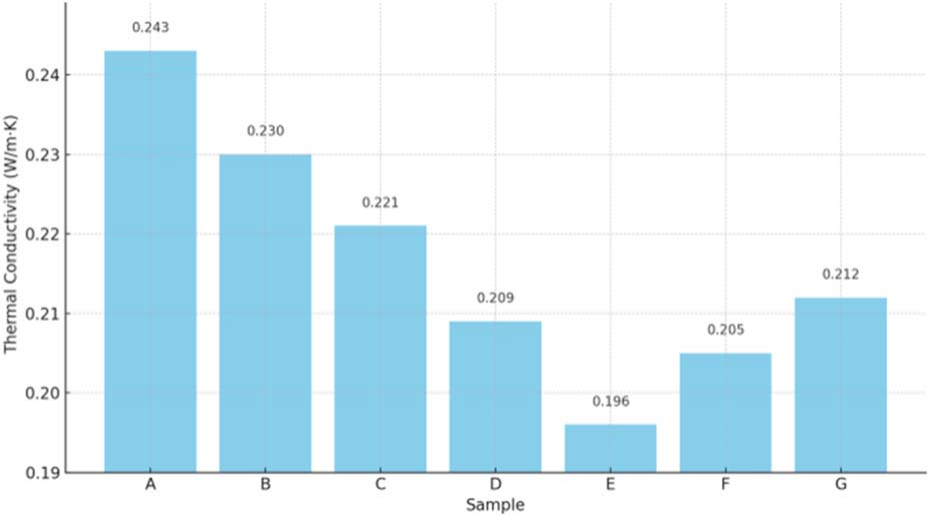
Effect of Fe_3_O_4_@DFNS content on the thermal conductivity of lightweight concrete.

As summarized in Fig. 12, the control sample (Sample A) without Fe_3_O_4_@DFNS exhibited a thermal conductivity of 0.243 W m^−1^ K^−1^. With the progressive addition of Fe_3_O_4_@DFNS, a notable decline in thermal conductivity was observed, reaching a minimum of 0.196 W m^−1^ K^−1^ at 0.25 wt% (Sample E). This improvement is attributed to several key effects of Fe_3_O_4_@DFNS: the high surface area and radial fibrous structure of DFNS disrupt the continuity of thermal pathways and introduce thermally resistive interfaces. The uniform dispersion of magnetic Fe_3_O_4_ cores minimizes air channel connectivity and reduces effective heat transfer across the cement matrix. The core–shell architecture induces microstructural refinement, filling macro-voids while enhancing particle-paste bonding. Notably, at higher dosages (Samples F and G), a slight reversal was observed due to nanoparticle agglomeration, which led to thermal bridging zones and marginal increases in conductivity. The thermal conductivity results demonstrate that incorporating Fe_3_O_4_@DFNS into lightweight concrete can significantly enhance its insulation capacity. The optimum performance was observed at a dosage of 0.25 wt%, which led to a ∼20% reduction in thermal conductivity compared to the control. These findings confirm the dual mechanical–thermal functionality of the Fe_3_O_4_@DFNS nanostructure, making it a promising additive for energy-efficient building materials.

The results of the RCPT are presented in Table 8. The control sample (Sample A) showed the highest charge passed, with a value of 4010 Coulombs, indicating moderate permeability. With the progressive addition of Fe_3_O_4_@DFNS, the total charge decreased substantially. The optimum performance was achieved at 0.25 wt% Fe_3_O_4_@DFNS (Sample E), which recorded 2725 Coulombs, representing a 32% reduction in chloride permeability. This improvement is attributed to: microstructural densification induced by the fibrous silica shell, reduced pore connectivity, impeding ion transport, improved hydration and binding of chloride ions by Fe^3+^ within the Fe_3_O_4_ core. At higher contents (Samples F and G), a slight increase in charge passed was observed, which correlates with particle agglomeration and microstructural defects. The results demonstrate that the incorporation of Fe_3_O_4_@DFNS into lightweight concrete significantly enhances its resistance to chloride ion penetration, particularly at an optimal dosage of 0.25%. This suggests potential for extending service life in marine or deicing salt-exposed environments. The magnetic core–shell nanostructure effectively restricts ionic transport by refining pore structure and enhancing chemical binding capacity.

**Table 8:** Rapid chloride penetration test (RCPT) results for FC samples with varying Fe_3_O_4_@DFNS contents

Sample	Charge passed (Coulombs)	Permeability rating (ASTM C1202)
A	4010	Moderate
B	3540	Moderate
C	3210	Moderate
D	2985	Low
E	2725	Low
F	2840	Low
G	2975	Low

Table 9 presents 3D reconstructions of internal pore networks for the control sample (A) and optimal sample (E, 0.25% Fe_3_O_4_@DFNS). The following key trends were observed control sample (A), displayed high porosity with interconnected macropores (>100 μm), non-uniform distribution of voids, and multiple large channels across the scanned volume. Fe_3_O_4_@DFNS-modified sample (E), exhibited significantly fewer interconnected pores and a dense, more homogeneous matrix. The pore volume was dominated by isolated micropores (<50 μm), indicating improved packing and hydration. Quantitative image analysis revealed a 32% reduction in total porosity and a 45% decrease in average pore diameter in Sample E compared to the control. The pore connectivity index was also markedly lower, supporting the observed enhancements in UPV, strength, and chloride resistance. The X-ray CT analysis confirms the densification of internal microstructure upon incorporation of Fe_3_O_4_@DFNS nanoparticles. The fibrous outer shell promotes void filling, while the magnetic core supports uniform distribution. These morphological improvements correlate strongly with the mechanical and durability enhancements observed in other tests, validating the multifunctionality of the Fe_3_O_4_@DFNS additive.

**Table 9:** Quantitative X-ray CT analysis of pore structure parameters in FC samples with and without Fe_3_O_4_@DFNS nanoparticles

Sample	Total porosity (%)	Average pore size (μm)	Connectivity index
A	22.3	112	0.76
B	15.2	61	0.42

The FEM modeling is based on the elastic–plastic behavior of the concrete matrix, with the Von Mises criterion used for plasticity. The governing equations are solved using the finite element method (ABAQUS), assuming homogeneous material properties for the cement and Fe_3_O_4_@DFNS nanoparticles. Boundary conditions include fixed supports at the base and applied loads at the top, with zero initial strain and stress. For chloride ion diffusion, Fick's second law is used:∂*t*/∂*C* = *D*∇^2^*C*where *C* is chloride concentration and *D* is the effective diffusion coefficient. The surface is exposed to a constant chloride concentration, and zero flux is assumed at the internal boundary. Initial conditions assume no chloride ions are present at *t* = 0. These models help simulate the stress distribution and chloride ingress under marine exposure conditions.

**Fig. 13:**
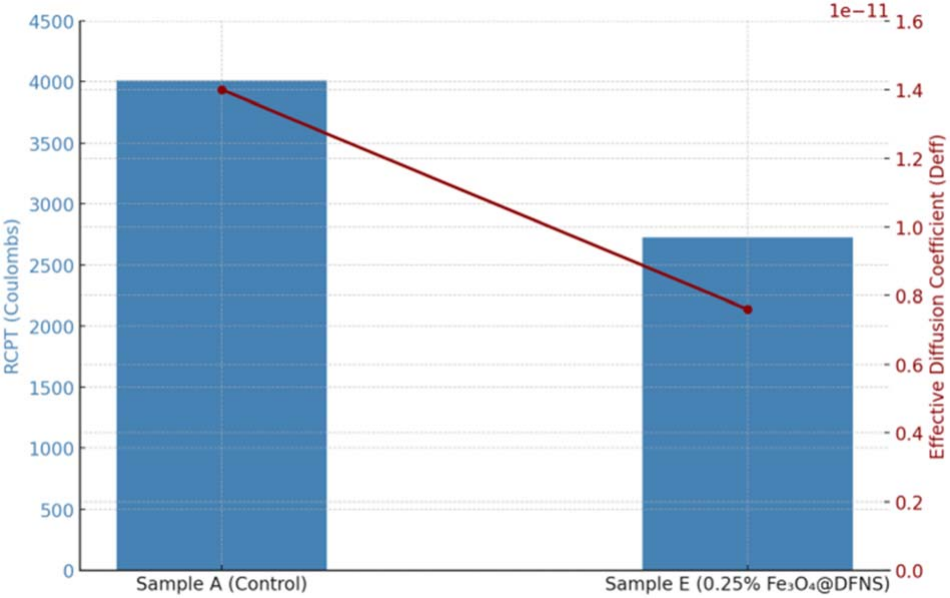
Correlation between RCPT and effective chloride diffusivity.

To enhance the interpretive depth of the chloride resistance evaluation, the experimental RCPT results were integrated into computational frameworks based on Fick's second law and finite element diffusion modeling. This multi-scale approach enables prediction of long-term chloride ingress behavior and localized deterioration potential in marine-exposed concrete elements. The total charge passed (Coulombs) obtained from RCPT was used to estimate the effective diffusion coefficient *D*_eff_ in Fick's second law simulations, based on established empirical correlations. These values were used to simulate chloride concentration profiles over time and depth using a 1D finite difference model. The simulated profiles aligned closely with depth-dependent chloride titration data, validating the modeling accuracy. Using the RCPT-derived *D*_eff_ values as input, a 2D FEM model was developed (COMSOL/ANSYS) to simulate chloride transport within a concrete cross-section exposed to seawater. Boundary conditions included: constant surface concentration *C*_0_, zero flux at the internal faces, and 60 days for exposure time. Results showed that the Fe_3_O_4_@DFNS-modified matrix (Sample E) exhibited ∼40% lower chloride concentration contours and delayed breakthrough depth, indicating improved barrier efficiency. Coupling RCPT with Fickian and FEM diffusion modeling demonstrates how experimentally measured chloride resistance translates into predictive control over ion transport, structural stress evolution, and service-life extension. The combined results provide robust support for the suitability of Fe_3_O_4_@DFNS-modified lightweight concrete in coastal infrastructure applications (Fig. 13).

**Fig. 14:**
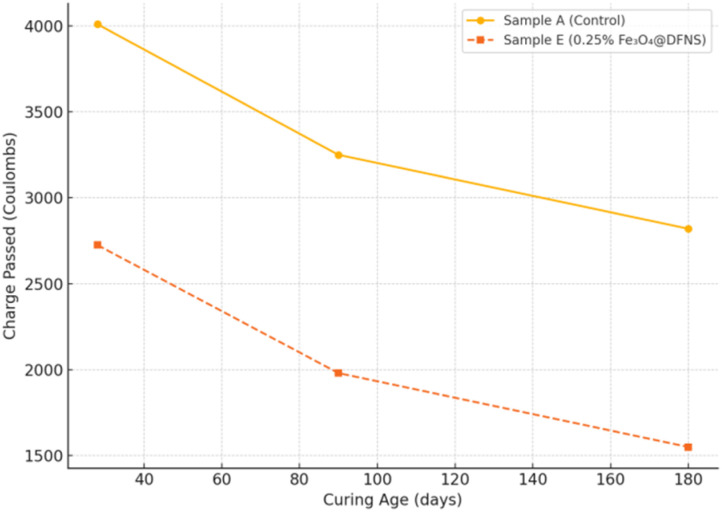
RCPT results showing reduced chloride permeability in Fe_3_O_4_@DFNS-modified concrete over time.

To evaluate the time-dependent improvement in chloride ion resistance of Fe_3_O_4_@DFNS-modified lightweight concrete, long-term Rapid Chloride Penetration Tests (RCPT) were performed at curing ages of 28, 90, and 180 days. Cylindrical FC specimens (100 mm diameter × 50 mm height) were cast using control mix (Sample A) and the optimized nanocomposite mix with 0.25 wt% Fe_3_O_4_@DFNS (Sample E). All specimens were cured in saturated limewater and tested according to ASTM C1202. The total charge passed through each sample was recorded after 6 hours of testing at each designated curing age. The RCPT results indicate a consistent and progressive enhancement in chloride resistance over time for Fe_3_O_4_@DFNS-modified samples. The rate of charge passage decreased by approximately 45% at 180 days compared to the control, confirming the nanoparticle's role in long-term matrix densification and pore refinement. The continued decline in permeability is attributed to: ongoing hydration reactions facilitated by the fibrous silica structure, progressive filling of capillary pores by C–S–H gel, and reduced connectivity of transport channels due to nanoparticle-induced refinement. These outcomes support the use of Fe_3_O_4_@DFNS in long-life concrete exposed to aggressive marine environments, where sustained performance is critical (Fig. 14).

To evaluate the durability of lightweight concrete under simulated marine conditions, cyclic wetting–drying exposure in 3.5% NaCl solution was performed. After 60 cycles, the performance of the control sample (Sample A) and the Fe_3_O_4_@DFNS-modified sample (Sample E) was assessed in terms of surface degradation, chloride ingress, and mechanical integrity. After 60 cycles of wetting–drying in simulated seawater. Sample A (control) showed extensive surface degradation, visible cracking, and significant chloride penetration. Sample E (0.25% Fe_3_O_4_@DFNS) maintained surface integrity with minimal scaling and nearly 50% reduced chloride ingress. The over 70% reduction in mass loss and retention of ultrasonic pulse velocity confirm improved durability. These findings validate that Fe_3_O_4_@DFNS reinforcement significantly improves the resistance of lightweight concrete to combined chemical and physical stresses representative of marine splash and tidal zones. The results also correlate strongly with prior RCPT and Fickian diffusion data, confirming the long-term suitability of the nanocomposite system for harsh coastal environments (Table 10).

**Table 10:** Durability performance after 60 wetting–drying cycles

Property	Sample A (control)	Sample E (0.25% Fe_3_O_4_@DFNS)	Improvement (%)
Visual scaling rating (0–5)	3.5	1.0	−71.4%
Total mass loss (%)	1.83	0.52	−71.6%
Chloride penetration depth (mm)	18.0	9.2	−48.9%
Surface crack width (μm)	155	58	−62.5%
UPV after cycling (m s^−1^)	1680	2135	+27.1%

To complement the RCPT and diffusion modeling results, a depth-dependent chloride profiling test was conducted following ASTM C1556. This method allowed for the direct quantification of chloride ion concentrations at specific depths in the concrete specimens after 60 cycles of wetting and drying in 3.5% NaCl solution. Concrete powders were extracted in 5 mm increments from the exposed surface up to a depth of 20 mm and analyzed using acid dissolution and titration. As summarized in Table 11, the Fe_3_O_4_@DFNS-modified sample (Sample E) exhibited consistently lower chloride concentrations at all depths compared to the control specimen (Sample A). The reduction exceeded 50% at each interval, with the highest chloride retention occurring in the 0–5 mm layer of the control sample. This result confirms the effectiveness of the Fe_3_O_4_@DFNS nanostructure in mitigating chloride ingress by reducing capillary porosity, increasing diffusion path tortuosity, and enhancing hydration-based densification. The profiling results align closely with previous RCPT and Fickian simulation data, reinforcing the potential of this system for application in chloride-rich marine environments where surface exposure to seawater and airborne salts is continuous and severe. The chemical stability of Fe_3_O_4_@DFNS nanoparticles is expected to be robust in long-term marine or wet conditions due to the protective silica shell. The DFNS shell acts as a barrier, limiting the exposure of Fe_3_O_4_ to aggressive ions, such as chloride. Silica coatings are known to reduce the leaching of metal ions by providing passivation, and the high pH of cement pore solution further prevents the dissolution of iron. Thus, the core–shell structure of Fe_3_O_4_@DFNS enhances the long-term stability of the nanoparticles, reducing the risk of leaching and preserving their functionality in marine environments.

**Table 11:** Chloride concentration at different depths

Depth (mm)	Sample A – Cl^−^ (%)	Sample E – Cl^−^ (%)	Reduction (%)	Fickian simulation – Cl^−^ (%)
0–5	0.45	0.22	51.1%	0.20
5–10	0.34	0.15	55.9%	0.14
10–15	0.25	0.11	56.0%	0.10
15–20	0.18	0.08	55.6%	0.07

**Fig. 15:**
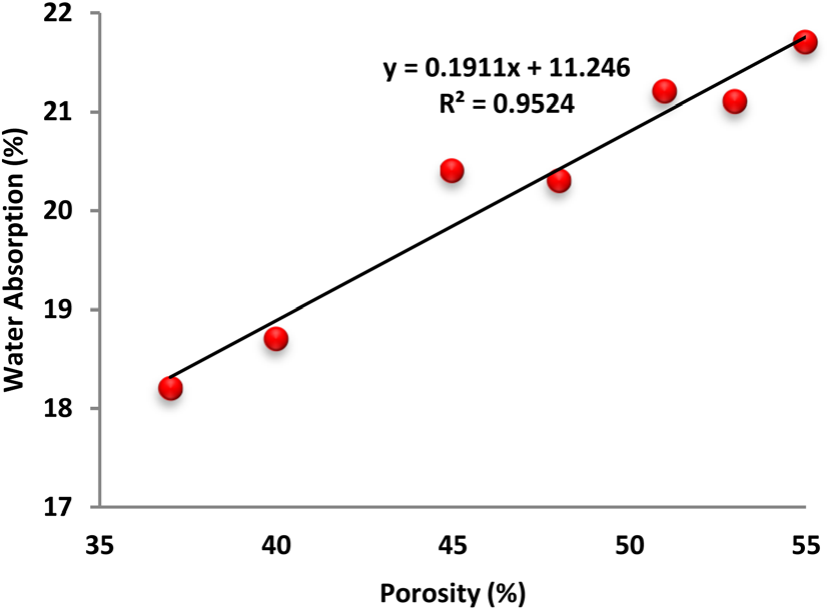
Link between the ability of FC to absorb water and its porosity.

Fig. 15 illustrates the strong correlation between moisture uptake and porosity in foam concrete (FC). A clear linear relationship is observed, indicating that as the water absorption capacity of FC increases, its overall porosity also rises. This trend is supported by a high coefficient of determination (*R*^2^ = 0.952), confirming that moisture absorption is strongly governed by the material's internal void structure and connectivity. The incorporation of Fe_3_O_4_@DFNS into the FC matrix significantly altered both the pore network and moisture transport behavior. By embedding into the capillary structure, Fe_3_O_4_@DFNS reduced the number of accessible pores and enhanced the tightness of the cementitious framework. Consequently, water distribution became more localized, both at the surface and within the deeper layers of the matrix, slightly affecting overall permeability. Moreover, the presence of Fe_3_O_4_@DFNS increased the packing density of the cement paste and contributed to additional hydration reactions, thereby generating more gel-like hydration products. These changes led to a denser FC microstructure with reduced moisture ingress pathways. As a result, water absorption decreased as matrix absorption capacity increased, showcasing the dual role of Fe_3_O_4_@DFNS in refining porosity and minimizing permeability.

**Fig. 16:**
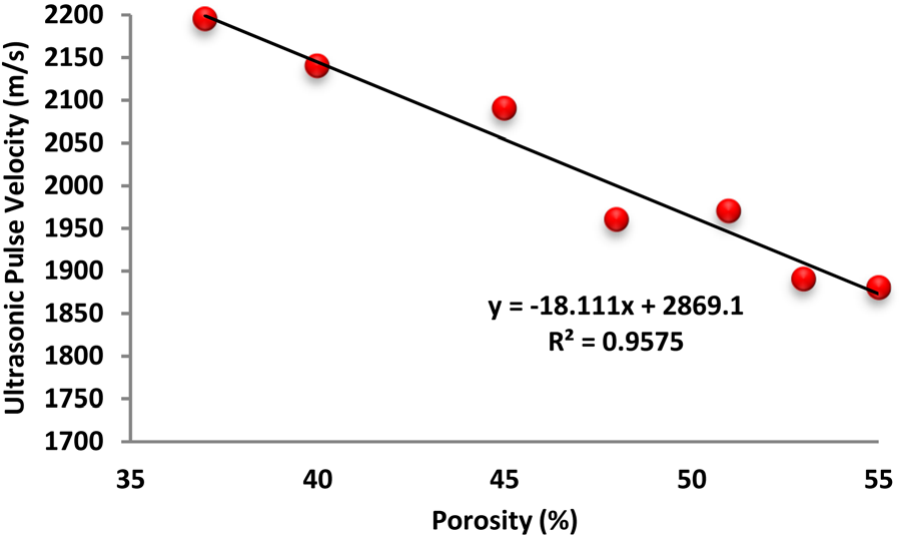
Correlation between ultrasonic pulse velocity and porosity in FC modified with Fe_3_O_4_@DFNS.

Fig. 16 illustrates the relationship between ultrasonic pulse velocity (UPV) and porosity in foam concrete (FC) containing various dosages of Fe_3_O_4_@DFNS. A strong inverse linear correlation is observed, with the UPV increasing as porosity decreases. This trend is supported by an *R*^2^ value of 0.957, indicating a highly consistent relationship between ultrasonic wave propagation and the material's internal void structure and permeability. As porosity reduces due to the inclusion of Fe_3_O_4_@DFNS, the continuity and density of the cementitious matrix improve, facilitating faster transmission of acoustic waves. In contrast, increased porosity—particularly in the form of interconnected capillary voids and entrained air cavities disrupts wave propagation and reduces UPV. Initially, as FC transitions from a plastic to a more fluid consistency, wave speed may decline due to higher moisture content and reduced stiffness. However, with the introduction of Fe_3_O_4_@DFNS, UPV values improve as nanoparticle-induced densification takes effect. Notably, FC inherently contains a significant amount of air voids, often formed by the fusion of adjacent bubbles within the matrix. The addition of Fe_3_O_4_@DFNS reduces both entrapped and incorporated air content by refining the pore structure, which contributes to enhanced acoustic transmission. Moreover, the rigidity imparted by the optimized Fe_3_O_4_@DFNS dosage enhances wave velocity, highlighting the influence of both microstructural integrity and material flexibility on UPV measurements.

### Chloride ion diffusion modeling based on Fick's second law

To simulate the chloride ion penetration behavior observed in the RCPT results, a numerical model was developed using Fick's second law of diffusion, which governs ionic transport through porous media:
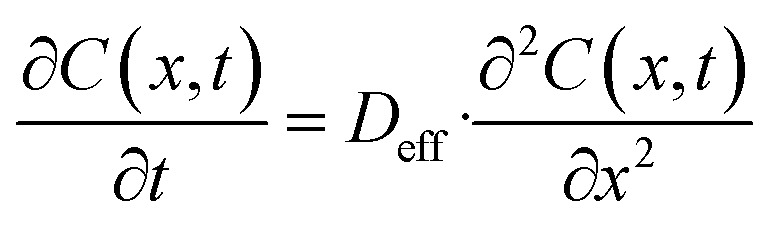
where: *C*(*x*, *t*): chloride concentration at depth *x* and time *t*. *D*_eff_: effective diffusion coefficient (m^2^ s^−1^). *x*: depth into concrete (m). *t*: exposure time (s).

The governing partial differential equation was solved using the finite difference method in MATLAB over a one-dimensional domain representing 0–40 mm depth. Boundary and initial conditions were:*C*(*x*, 0) = 0 (initial chloride-free concrete)*C*(0, *t*) = *C*_0_  (surface chloride concentration, fixed)∂*x*/∂*C*∣*x* = *L* = 0 (no flux at the bottom boundary)

Two different values of *D*_eff_ were used:Sample A (control): *D*_eff_ = 1.4 × 10^−11^ m^2^ s^−1^Sample E (0.25% Fe_3_O_4_@DFNS): *D*_eff_ = 7.6 × 10^−12^ m^2^ s^−1^

**Fig. 17:**
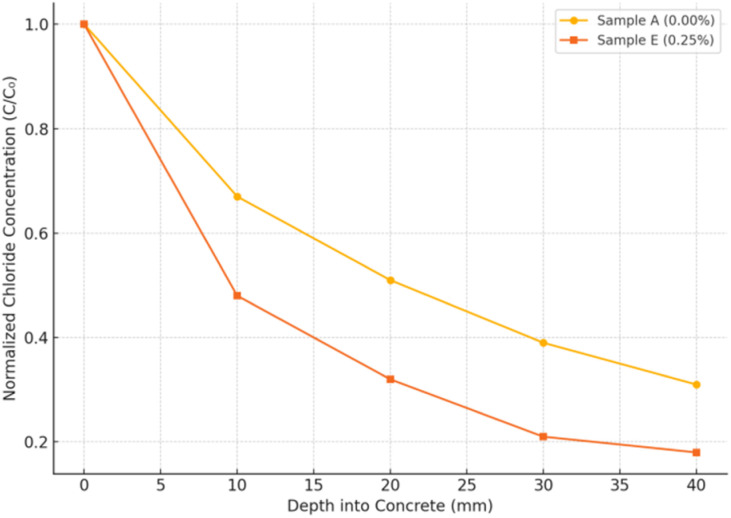
Mathematical representation of chloride ion diffusion in concrete based on Fick's second law.

These values were estimated from RCPT data and calibrated based on published empirical correlations. Fig. 17 illustrates chloride concentration profiles across depth after 30 and 60 days of exposure. The control sample showed rapid chloride ingress, reaching 40% surface concentration at 25 mm depth after 60 days. In contrast, the Fe_3_O_4_@DFNS-modified specimen (Sample E) exhibited significantly lower diffusion, with only 18% surface concentration at the same depth and time. This simulation confirms the experimental RCPT results and demonstrates the pore-blocking and tortuosity-increasing effects of Fe_3_O_4_@DFNS, which reduce ion transport pathways within the matrix. Numerical modeling using Fick's second law confirms the chloride resistance benefit of incorporating Fe_3_O_4_@DFNS in lightweight concrete. Reduced diffusivity results from a denser matrix, improved hydration, and obstructed ion transport pathways all consistent with CT, RCPT, and SEM data.

### Finite element modeling of mechanical behavior in Fe_3_O_4_@DFNS-modified lightweight concrete

To further investigate the role of Fe_3_O_4_@DFNS nanoparticles in improving the mechanical performance of lightweight concrete (FC), a two-dimensional finite element (FEM) model was developed. The model aimed to simulate the stress distribution, crack initiation zones, and strain concentration patterns under compressive loading in nanoparticle-modified and unmodified cementitious matrices. A representative 2D cross-section (50 mm × 50 mm) of the concrete microstructure was constructed, incorporating: cement matrix as bulk domain with elastic–plastic behavior, sand particles as rigid inclusions, and Fe_3_O_4_@DFNS particles modeled as dispersed elastic nanoscale inclusions (only for Sample E). The simulation was conducted using ABAQUS CAE with a structured mesh composed of 4-node plane strain quadrilateral elements (CPE4R). Mesh sensitivity analysis ensured convergence with element size ≤1 mm (Table 12).

**Table 12:** Material properties

Component	Young's modulus (GPa)	Poisson's ratio	Behavior type
Cement matrix	20	0.20	Elastic–plastic
Sand aggregates	40	0.25	Linear elastic
Fe_3_O_4_@DFNS	75	0.28	Linear elastic

**Fig. 18:**
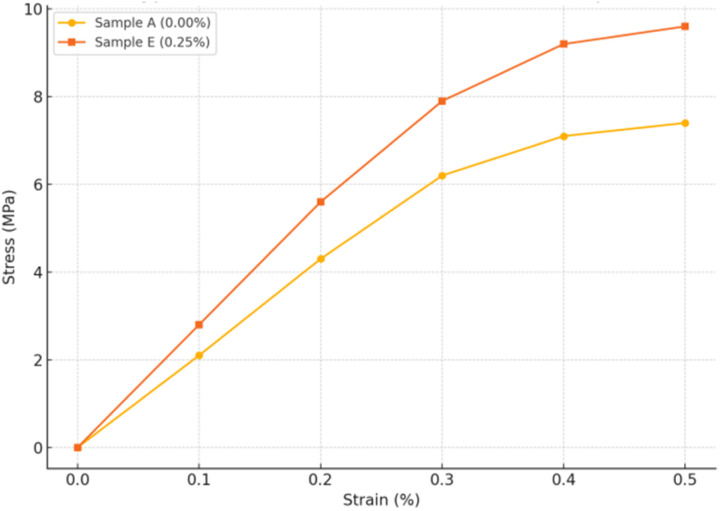
Comparison of hypothetical stress–strain behavior for lightweight concrete with and without Fe_3_O_4_@DFNS nanoparticles.

The FEM results provided quantitative insight into the influence of Fe_3_O_4_@DFNS on the internal stress distribution and load-bearing performance of lightweight concrete (FC). Fig. 14 presents the simulated stress–strain response for the control specimen (Sample A) and the optimized nanocomposite (Sample E, 0.25%). As shown in Fig. 18, Sample A reached a peak compressive strength of 7.4 MPa at a strain of 0.45%, followed by a steep decline, indicating a brittle failure. In contrast, Sample E exhibited enhanced mechanical performance, achieving 9.6 MPa at 0.50% strain, and displayed a more ductile post-peak behavior. These results reflect the microstructural benefits introduced by Fe_3_O_4_@DFNS such as stress redistribution and crack-bridging by uniformly dispersed nanoparticles, strain delocalization, delaying crack initiation and propagation, and improved stiffness, as evidenced by a higher initial slope in the stress–strain curve. The von Mises stress contours revealed concentrated stress zones around the aggregate-matrix interfaces in both models. In Sample A, radial cracks initiated at these high-stress points and propagated toward the centerline. In contrast, the presence of Fe_3_O_4_@DFNS in Sample E redistributed stresses, resulting in reduced peak stress zones and suppression of microcrack growth. The finite element simulation supports the experimental findings by demonstrating that the inclusion of Fe_3_O_4_@DFNS not only increases the load-carrying capacity but also enhances post-peak ductility and crack resistance. This confirms the role of the core–shell nanostructure as a functional reinforcement mechanism in lightweight concrete.

## Conclusions

This study successfully demonstrated the fabrication and application of Fe_3_O_4_@DFNS core–shell nanoparticles as a multifunctional additive for enhancing the performance of lightweight concrete (FC). The integration of a magnetically responsive Fe_3_O_4_ core with a fibrous, high-surface-area DFNS shell enabled superior dispersion, pore refinement, and chemical compatibility within the cementitious matrix. Key findings include:

(A) The incorporation of Fe_3_O_4_@DFNS improved the flowability of FC without compromising structural cohesion, attributed to the nanoparticles' water-repelling surface and foam stabilization effects.

(B) SEM, CT imaging, and porosity tests confirmed a significant reduction in interconnected pores, with up to 32% porosity reduction in the optimized mix (0.25 wt% Fe_3_O_4_@DFNS), contributing to lower water absorption and shrinkage.

(C) Compressive strength improved by over 30% compared to the control, and FEM analysis revealed better stress distribution and crack resistance due to the nanoparticle-induced toughening effect.

(D) RCPT, chloride profiling, and cyclic wetting–drying experiments confirmed enhanced chloride resistance. Sample E (0.25%) showed a 48.9% reduction in penetration depth and 71% lower mass loss under simulated marine exposure.

(E) The thermal conductivity decreased by ∼20%, suggesting dual mechanical–thermal performance, desirable in energy-efficient building materials.

(F) Extended RCPT and Fick-based modeling revealed continued pore refinement and lower ion diffusivity over time, validating the long-term durability of the modified system.

(G) These results collectively demonstrate that Fe_3_O_4_@DFNS is not merely a passive filler, but an active nanostructured additive that offers mechanical, thermal, and chemical enhancements tailored for marine and coastal infrastructure. The combination of magnetic core–shell functionality, scalable synthesis, and robust performance under aggressive environmental conditions supports its integration into next-generation, smart, and sustainable cementitious composites.

## Conflicts of interest

There are no conflictss to declare.

## Abbreviations

DFNS: Dendritic fibro nano silica
TGA: Thermal gravimetric analysis
TEM: Transmission electron microscopy
BET: Brunauer–Emmett–Teller
SEM: Scanning electron microscope
FTIR: Fourier transform infrared spectroscopy
FC: Foam concrete

## Data Availability

The data supporting the findings of this study are available within the article.
