# Correction: Structuring lightweight concrete with core–shell Fe_3_O_4_@DFNS nanoparticles: a path to smart and durable cementitious materials

**DOI:** 10.1039/d5ra90124c

**Published:** 2025-10-31

**Authors:** Seyed Ali Haji Seyed Abotorabi, Amin Honarbakhsh, Rahele Zhiani, Seyed Mojtaba Movahedifar, Mehdi Nobahari

**Affiliations:** a Department of Civil Engineering, Ne.C., Islamic Azad University Neyshabur Iran amin_honarbakhsh@iau.ac.ir; b Department of Chemistry, Ne.C., Islamic Azad University Neyshabur Iran Zhiani@iau.ac.ir r_zhiani2006@yahoo.com

## Abstract

Correction for ‘Structuring lightweight concrete with core–shell Fe_3_O_4_@DFNS nanoparticles: a path to smart and durable cementitious materials’ by Seyed Ali Haji Seyed Abotorabi *et al.*, *RSC Adv.*, 2025, **15**, 34079–34093, https://doi.org/10.1039/D5RA04695E.

The authors regret that the name of one of the authors (Seyed Ali Haji Seyed Abotorabi) was incompletely shown in the original article. The corrected author list is as shown here.

The Royal Society of Chemistry apologises for these errors and any consequent inconvenience to authors and readers.

